# Determinants of age‐appropriate breastfeeding, dietary diversity, and consumption of animal source foods among Indonesian children

**DOI:** 10.1111/mcn.12889

**Published:** 2019-10-02

**Authors:** Susy K. Sebayang, Michael J. Dibley, Erni Astutik, Ferry Efendi, Patrick J. Kelly, Mu Li

**Affiliations:** ^1^ Department of Biostatistics and Population Studies Universitas Airlangga, Banyuwangi Campus Banyuwangi Indonesia; ^2^ Sydney School of Public Health University of Sydney Sydney New South Wales Australia; ^3^ Department of Epidemiology Universitas Airlangga, Banyuwangi Campus Banyuwangi Indonesia; ^4^ Faculty of Nursing Universitas Airlangga Surabaya Indonesia

**Keywords:** breastfeeding, dietary diversity, animal source food, child feeding, Indonesia

## Abstract

Global child feeding practices remain suboptimal. In this study, we assess the determinants of age‐inappropriate breastfeeding, dietary diversity, and consumption of 3+ types of animal source foods (ASFs) using 11,687 observations from combined data from the Indonesian Demographic Health Survey of 2012 and 2017. We used linear and logistic regression after adjusting for the complex sampling design. Child's age and quality of antenatal care (ANC) were associated with all outcomes. Socio‐economic status and labour force participation were positively associated with higher dietary diversity score, ASF consumption, and age‐inappropriate breastfeeding. More ANC visits and having consultation at ANC were associated with more dietary diversity. Higher women's knowledge level was associated with more dietary diversity and consuming more ASF. Compared with western Indonesia, more children in eastern Indonesia were age‐inappropriately breastfed and had lower dietary diversity. The Indonesian government needs to develop programmes to improve child feeding particularly in eastern Indonesia, focusing on improving dietary diversity and ASF consumption in poorer households and on prolonging breastfeeding in richer households. Women's labour force participation should be encouraged, but programmes for working mothers are also needed to support continued breastfeeding and to express breast milk. ANC and postnatal programmes need improved consultation sessions for child feeding.

**Key messages**

- Child's age and quality of ANC were associated with age‐inappropriate breastfeeding, dietary diversity, and ASF consumption.
- Socio‐economic status and labour force participation were positively associated with age‐inappropriate breastfeeding, dietary diversity, and ASF consumption.
- Government programs are needed to improve child feeding in eastern Indonesia and some western provinces, to improve dietary diversity and ASF in poorer households, and to prolong breastfeeding duration in richer households.
- National programs that support continued breastfeeding and expressing breast milk for working mothers are required.
- To improve the quality of ANC programs, consultation sessions to discuss appropriate child feeding practices need strengthening.

## 1 INTRODUCTION

Appropriate child feeding practice from birth contributes to improvement in child survival, achieving growth and development potential, prevention of micronutrient deficiencies, future morbidity, and obesity in adulthood (Begin & Aguayo, 2017). The link between stunting and reduced cognitive abilities (de Onis & Branca, 2016), education opportunities, and up to 7% reduction in per capita income (Galasso, Wagstaff, Naudeau, & Sheekar, 2016) has generated a lot of research interests on the role of appropriate child feeding on reducing stunting. Some studies show negative associations between dietary diversity (Mahmudiono, Sumarmi, & Rosenkranz, 2017; Marriott, White, Hadden, Davies, & Wallingford, 2012; Rah et al., 2010) and stunting. By feeding five or more food groups to under‐five children would avert 13% of stunting in this age group (Krasevec, An, Kumapley, Begin, & Frongillo, 2017). Higher dietary diversity is associated with higher calcium adequacy (Muslimatun & Wiradnyani, 2016), which may link dietary diversity with child physical growth. Similarly, higher consumption of animal source food (ASF; Headey, Hirvonen, & Hoddinott, 2018; Krasevec et al., 2017) or higher household expenditure on ASF (Sari et al., 2010) is also associated with a lower rate of stunting. Moreover, ASF consumption is also positively associated with adequacy of protein and micronutrient intakes, especially Vitamin A, calcium, and zinc (Muslimatun & Wiradnyani, 2016), which are not only important for child growth but also for child development.

In addition to stunting, appropriate infant and young child feeding can reduce child mortality. Globally, scaling up exclusive and continued breastfeeding to up to 90% could prevent 823,000 deaths in under‐five children annually (Victora et al., 2016). Not breastfeeding is associated with reduced cognition and economic losses of approximately $302 billion annually (Rollins et al., 2016). In Indonesia, the cost of not breastfeeding reached $1,343.70 million from cognitive losses alone (Rollins et al., 2016) and $118.62 million from treating diarrhoea and respiratory disease annually (Siregar, Pitriyan, & Walters, 2018), the highest in south‐east Asia. A 10% point increase in exclusive breastfeeding and continued breastfeeding rates up to 1 or 2 years could reduce costs of treatment for child morbidity by $350 million in the United States, United Kingdom, China, and Brazil combined (Rollins et al., 2016). In south‐east Asia, the economic benefits of breastfeeding through higher cognition and potentially higher earnings were estimated to be approximately $1.6 billion per year (Walters et al., 2016).

Globally, there were 151 million under‐five children stunted in 2017 (United Nations, 2018). Less than 30% of children aged 6–23 months in the world meet the minimum dietary diversity, with only 42% of children in this age group consumed dairy products, 28% consumed ASF, and 17% consumed eggs (J. M. White, Begin, Kumapley, Murray, & Krasevec, 2017). In Indonesia, the prevalence of exclusive breastfeeding in the first 6 months is only 37.3%, and 47% of children 6–23 months consume four out of seven food groups (Indonesian National Institute of Health Research and Development‐Ministry of Health, 2018). With currently 31% of Indonesian under‐five children and 30% of under‐two children stunted (Indonesian National Institute of Health Research and Development‐Ministry of Health, 2018), Indonesia is set to miss its target of reducing stunting among under‐two children to 28% (Ministry of Health of the Republic of Indonesia, 2015). However, Indonesia is currently focusing efforts to reduce stunting through the national strategy to accelerate stunting prevention, which, if fully implemented, will potentially reduce by two million the number of stunted children in this country (Rokx, Subandoro, & Gallagher, 2018). Thus, it is important to understand the determinants of appropriate child feeding in Indonesia to provide evidence to support stunting prevention strategies through provision of adequate nutrition.

Studies have shown that dietary diversity was positively associated with the child's age (Iqbal, Zakar, Zakar, & Fischer, 2017), maternal education, socio‐economic status (Marinda, Genschick, Khayeka‐Wandabwa, Kiwanuka‐Lubinda, & Thilsted, 2018; Patel et al., 2012; Senarath et al., 2012; Solomon, Aderaw, & Tegegne, 2017), mother's working status (Solomon et al., 2017), women's empowerment (Ickes, Wu, Mandel, & Roberts, 2018), and the number of antenatal care (ANC) visits (Patel et al., 2012). There are reports of differences in consumption of ASF by residence and wealth status and child's age (J. M. White et al., 2017). A review also concluded that maternal autonomy might be an important factor in improving child feeding and nutritional status (Carlson, Kordas, & Murray‐Kolb, 2015). In Indonesia, exclusive breastfeeding is reported to be associated with socio‐economic status and working parents (Titaley, Loh, Prasetyo, Ariawan, & Shankar, 2014). However, more studies from Indonesia are needed to guide programmes to improve child feeding practices in this country. This study, therefore, assesses a more complex set of determinants of age‐inappropriate breastfeeding, dietary diversity, and consumption of ASF in children 0–23 months in Indonesia using nationally representative survey data.

## 2 METHODS

### 2.1 Data

Indonesian Demographic Health Surveys (IDHS) are conducted every 5 years. We used data from the two most recent surveys (2012 and 2017), which we combined for our analyses.

IDHS employs a complex sampling design with stratification by regions and urban/rural areas before sampling the households. The IDHS country reports (available at http://www.dhsprogram.com) describe in detail the sampling methods used in the surveys. Institutionalised review board in ICF, as the DHS survey implementing agency, and institutionalised review board in host countries approved the survey protocols. All participants provided informed consent before data collection. We obtained the data in this study from the DHS website (http://www.dhsprogram.com) and the Indonesian Coordinating Board for National Family Planning website (https://sdki.bkkbn.go.id). This study is a secondary analysis of de‐identified data, and thus, no further consent was needed.

All women aged 15–49 years in the selected households were eligible for interview by the trained enumerators. In the surveys, women with under‐five children were interviewed to collect data about their children. We analysed dietary data of the youngest child aged 0–23 months born in the 5 years preceding the survey, who was alive and lived with their mother.

### 2.2 Key variables and measurements

Infant feeding practices were assessed using three indicators: age‐inappropriate breastfeeding, dietary diversity, and number of types of ASF consumed with the following definitions:

1. We defined age‐inappropriate breastfeeding as not breastfeeding the child for his/her age as recommended by World Health Organization. Breastfeeding is considered appropriate for the age when infants aged 0–5 months receive only breast milk and children aged 6–23 months receive solid, semisolid, or soft foods in addition to breast milk within 24 hr before the survey (World Health Organization, 2010). For ease of programmatic interpretation, this study analysed the odds of age‐inappropriate breastfeeding.
2. Dietary diversity: The number of food groups consumed within 24 hr prior to the survey from a total of eight food groups, which included (a) breast milk; (b) grains, roots, and tubers; (c) legumes and nuts; (d) dairy products; (e) flesh foods; (f) eggs; (g) Vitamin‐A rich fruits and vegetables; and (h) other fruits and vegetables (Technical Expert Advisory Group on Nutrition Monitoring, 2017). This study analysed the increase in the mean dietary diversity score.
3. Number of types of ASF consumed from a possible six groups from the following list: (a) breast milk, fresh milk, powdered milk, and baby formula; (b) other processed milk product such as yogurt and cheese; (c) eggs; (d) meats (beef, pork, lamb, and chicken); (e) organs (liver, heart, and other organs); and (f) fish or shellfish. On the basis of the distribution of the variety of ASF types by the number of types consumed, a child is considered to have an adequate number of ASF if fed three or more types of ASF in the 24 hr before the interview. This study analysed the odds of consuming 3+ types of ASF.

We tested a list of determinants previously reported to be associated with age‐inappropriate breastfeeding, dietary diversity, and consumption of 3+ types of ASF (analytical framework is available on Figure S1):

1. Child factors: gender, child's age (0–5 months, 6–8 months, 9–11 months, 12–17 months, and 18–23 months), and birth order (first child, second child, and third or later child).
2. Demographic factors: mother's age (15–19 years, 20–14 years, 25–29 years, 30–34 years, 35–39 years, and 40+ years), age difference between mother and father (older than father, 0–4 years, 5–7 years, and >7 years), father's education (did not complete primary education or less, completed primary or some secondary school, and completed secondary school or higher), father's occupation (agricultural and nonagricultural), residence (urban or rural), and region (west or east Indonesia). West Indonesia covered all provinces in and around Sumatera and Java island, and remaining provinces are in East Indonesia. The Indonesian government usually includes Kalimantan as a part of west Indonesia. However, because two of four provinces in Kalimantan were among the worst half in age‐inappropriate breastfeeding and dietary diversity, we included Kalimantan as east Indonesia in our analysis. We reanalysed the final models using Kalimantan as west Indonesia to assess the difference in the estimates.
3. Household factors: wealth quintiles (lowest, second, third, fourth, and highest), number of means of transport owned (did not own any, owned one, and owned ≥2 means of transport), size of agricultural land owned (did not own land, owned <3 ha, 3–9 ha, and 10+ ha), number of children under five (<2 or ≥2). We calculated the wealth quintiles by combining the two surveys and using principal component analysis of household ownership of an inventory of assets and facilities, which included electricity, radio, television, telephone, mobile phones, refrigerator, bank account, number of members of household per sleeping room, source of water, type of toilets, materials for floor and roof, and type of cooking fuel. We included the number of means of transport owned as a proxy indicator for access to food markets and ownership of agricultural land as a proxy for food availability.
4. Women's access to and contact with health care providers, including the number of ANC visits attended, the quality of the ANC, ANC consultation, and the provision of information about the source of care for pregnancy complications. For age‐inappropriate breastfeeding, we also evaluated assisted delivery as a determinant. We calculated an indicator for ANC quality by adding the number of ANC health services the woman received during her pregnancy, which included weight, height, and blood pressure measurement, abdominal exam, urine and blood tests, tetanus injection, and received or bought iron supplements. We categorised the quality score into ≤3, 4–6, and >6 types of ANC services mothers received in her last pregnancy. We also added antenatal consultation and being told about the source of care for pregnancy complications (no consultation, received either consultation or the information about the source of care for pregnancy complication, and received both) as a separate indicator for ANC quality. We defined assisted delivery as a delivery that was assisted by a medical doctor, obstetricians, nurse, midwife, or village midwife.
5. Women's empowerment factors: labour force participation, disagreement to justification toward wife beating, decision‐making power, and women's knowledge level. Women's empowerment factors were calculated by combining the data of the two surveys and using principal component analysis of 17 indicators following a previous method (Sebayang, Efendi, & Astutik, 2019). The 17 indicators included indicators for labour force participation (women's working status and income), her disagreement with several justification for wife beating, her decision‐making power in the household over women's own health care, household purchases, visiting family, and husband's earnings and her knowledge level (formal education level and access to media).

### 2.3 Statistical analysis

To provide a programmatic evaluation of the child feeding status and progress over time, we graphed the percentage of age‐inappropriate breastfeeding, the mean dietary diversity, and the percentage of consumption of 3+ types of ASF by province, overall, and by the year of the survey. We used logistic regression to determine associations between the covariates and age‐appropriate breastfeeding and consumption of ASF. The dietary diversity score was normally distributed, and thus, the association between the covariates and dietary diversity was assessed using multiple linear regression. Covariates univariably associated with a *P* value <.25 were included in the initial multivariable regression models. Final models were derived using backward elimination of variables, and we only retained those variables with *P* value <.01 in the final model. We used a lower significance level due to the large sample size, and our intention only to detect factors that were strongly associated with the outcome. Survey year and region were kept in the model regardless of their significance. We replaced region with province in the model to show details of the association by province. In all our analyses, we categorised North Kalimantan, a new province in IDHS 2017, with East Kalimantan, as was the case in IDHS 2012.

All analyses were conducted using stata 15 using the svy commands to adjust for the complex sampling design.

## 3 RESULTS

**Figure 1:**
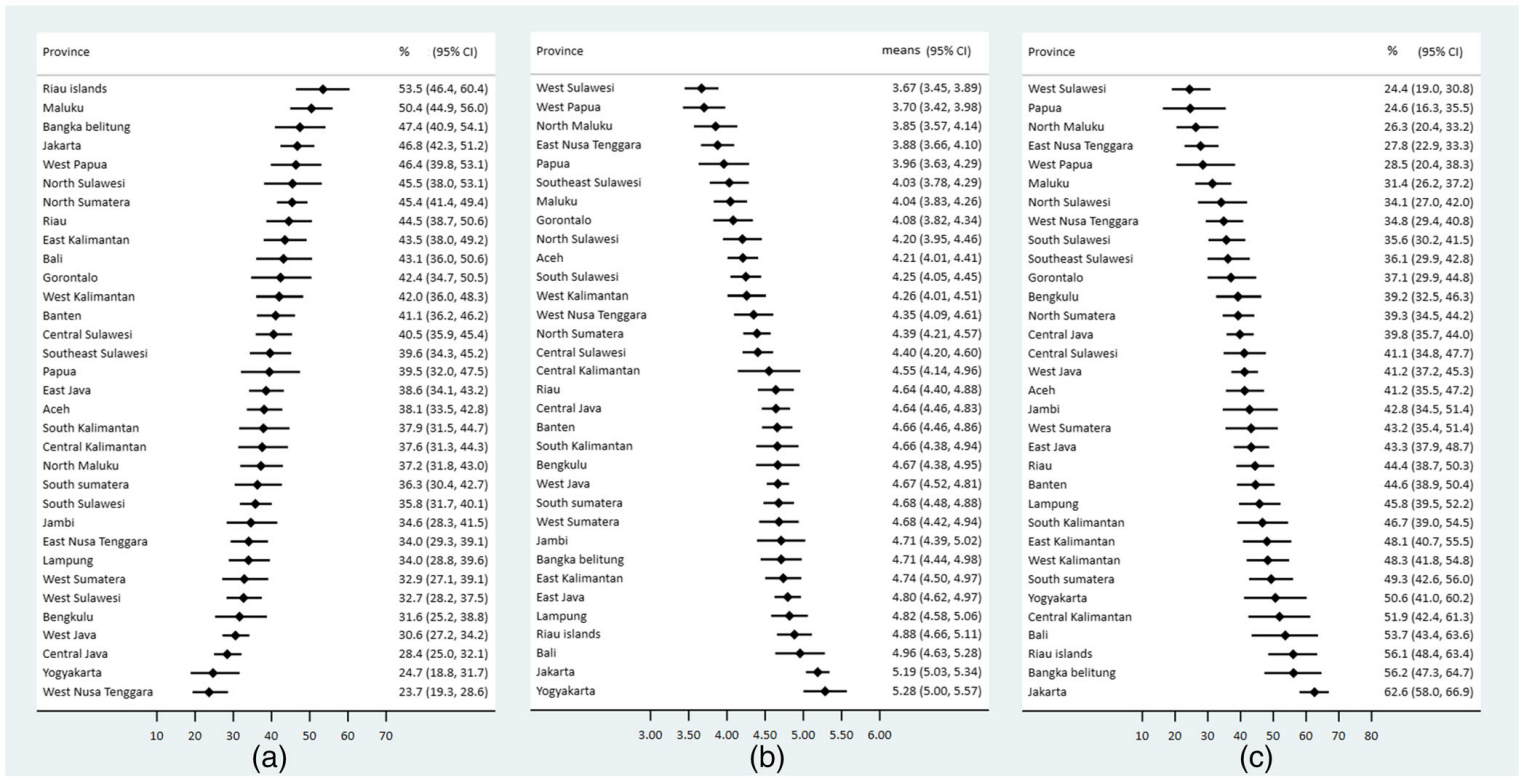
Child feeding in Indonesia in 2012–2017: (a) Percentage of age‐inappropriate breastfeeding by province; (b) Means of dietary diversity scores by province in Indonesia; and (c) Percentage of 3+ types of animal source food consumption

There were 11,687 children aged 0–23 months in the sample. Most children were aged 12–17 months (26%) and most had a mother who had a husband with a nonagricultural job (65%), half lived in rural areas, 68% belonged to households that did not own agricultural land, and 67% lived at home with <2 under‐five children (Table 1). There were 4,221 (36.1%) age‐inappropriately breastfed children aged 0–23 months, with a range from 23.7% in West Nusa Tenggara to 53.5% in Riau Islands. There were 3,490 (39.3%) children who met minimum dietary diversity, with the number of types of foods ranging from 3.67 types in West Sulawesi to 5.28 in Yogyakarta. Moreover, there were 3,775 (42.5%) children fed three or more types of ASF (from 24.4% in West Sulawesi to 62.6% in Jakarta; Table 1 and Figure 1a–c). From 2012 to 2017, West Nusa Tenggara and Yogyakarta had the lowest percentage of age‐inappropriately breastfed children, whereas Riau Islands and Maluku had the highest, and Bangka Belitung was the most improved, with a 40% reduction between surveys (Figure S2). Bangka Belitung was also the most improved province with a 20% point increase in dietary diversity score between surveys, and Papua was the worst performing province with a 17% point reduction in dietary diversity between surveys (Figure S3). Bali was the best performing province with 76% increase in child consumption of ASF, whereas Papua was again the worst performing with 51% reduction of ASF from 2012 to 2017 (Figure S4).

**Table 1:** Characteristics of last‐born children in combined 2012 and 2017 surveys age 0–23 months (population *N* = 11,687) and age 6–23 months (population *N* = 8,878)

Variables	Age (0–23 months)		Age (6–23 months)	
	*n*	% or mean ± *SD*	*n*	% or mean ± *SD*
Survey year				
2012	5,827	49.9	4,420	49.8
2017	5,860	50.1	4,458	50.2
Age‐appropriate breastfeeding				
No	4,221	36.1		
Yes	7,465	63.9		
Minimum dietary diversity				
No			5,388	60.7
Yes			3,490	39.3
Type of animal source food consumed				
<3			5,102	57.5
3+			3,775	42.5
Child factors				
Gender				
Male	5,988	51.2	4,579	51.6
Female	5,698	48.8	4,298	48.4
Child's age				
0–5 months	2,809	24.0		
6–8 months	1,487	12.7	1,487	16.8
9–11 months	1,569	13.4	1,569	17.7
12–17 months	3,040	26.0	3,040	34.2
18–23 months	2,781	23.8	2,781	31.3
Birth order				
First child	4,152	35.5	3,132	35.3
Second child	4,038	34.6	3,061	34.5
3+	3,496	29.9	2,685	30.2
Demographic factors				
Mother's age				
15–19 years	565	4.8	337	3.8
20–24 years	2,562	21.9	1,867	21.0
25–29 years	3,214	27.5	2,458	27.7
30–34 years	2,881	24.7	2,240	25.2
35–39 years	1,805	15.4	1,430	16.1
40+ years	660	5.6	546	6.2
Age difference between mother and father				
Woman older than man	2,011	17.2	1,534	17.3
0–4 years	4,679	40.0	3,590	40.4
5–7 years	2,562	21.9	1,945	21.9
>7 years	2,435	20.8	1,809	20.4
Father's educational attainment				
Incomplete primary education/none	894	7.7	686	7.7
Complete primary or some secondary	5,362	45.9	4,103	46.2
Completed secondary or higher	5,431	46.5	4,089	46.1
Father's occupation				
Agricultural	4,131	35.3	3,129	35.2
Nonagricultural	7,556	64.7	5,749	64.8
Residence				
Urban	5,782	49.5	4,419	49.8
Rural	5,905	50.5	4,458	50.2
Region				
West	9,120	78	6,959	78.4
East	2,566	22	1,918	21.6
Household factors				
Wealth quintiles				
Lowest	2,015	17.2	1,520	17.1
Second	2,219	19.0	1,641	18.5
Third	2,449	21.0	1,898	21.4
Fourth	2,527	21.6	1,913	21.5
Highest	2,477	21.2	1,906	21.5
Number of means of transport owned				
None	1,662	14.2	1,270	14.3
One means	5,333	45.6	4,048	45.6
Two or more	4,691	40.1	3,559	40.1
Area of agricultural land				
Did not own agricultural land	7,898	67.6	6,043	68.1
<3 ha	1,184	10.1	879	9.9
3–9 ha	1,129	9.7	825	9.3
10+ ha	1,476	12.6	1,130	12.7
Number of children under five				
<2	7,921	67.8	6,238	70.3
Two or more	3,765	32.2	2,640	29.7
Health care factors				
Number of ANC visit		8.13 ± 3.55		8.18 ± 3.49
Quality of ANC				
1–3 types of service	788	6.7	577	6.5
4–6 types of service	6,451	55.2	4,883	55.0
7–8 types of service	4,448	38.1	3,418	38.5
ANC: had consultation and told about care for complication				
Not had consultation nor told about care	1,250	10.7	963	10.9
Had either one	4,390	37.6	3,309	37.3
Had both	6,047	51.7	4,605	51.9
Delivery assistant				
Untrained	1,132	9.7	893	10.1
Trained	10,554	90.3	7,985	89.9
Women's empowerment factors^a^				
Mother's labour force participation				
Low	4,090	35.0	3,106	35.0
Medium	3,799	32.5	2,877	32.4
High	3,798	32.5	2,895	32.6
Mother's Disagreement Towards Wife Beating				
Low	3,657	31.3	2,792	31.5
Medium	3,932	33.6	3,022	34.0
High	4,098	35.1	3,064	34.5
Mother's decision‐making power				
Low	3,935	33.7	2,971	33.5
Medium	3,884	33.2	3,002	33.8
High	3,867	33.1	2,905	32.7
Mother's knowledge level				
Low	4,071	34.8	3,043	34.3
Medium	4,030	34.5	3,137	35.3
High	3,586	30.7	2,698	30.4

Abbreviations: ANC, antenatal care; *SD*, standard deviation.

a Women's empowerment factors were calculated by combining the data of the two surveys and using principal component analysis of 17 indicators following a previous method (Sebayang et al., 2019). Six variables indicated labour force participation: work in the last 12 months, for whom the woman worked, women's occupation type, types of payment, worked all year, and earned more than the husband. Women's disagreement with justification for wife beating was assessed with five reasons: neglecting children, going out without husband's permission, arguing with husband, refusing sex, and burning food. Decision‐making power was derived from household decision makers for women's own health care, household purchases, visiting family, and husband's earnings. The women's knowledge component included formal educational level and access to media (newspaper, radio, and television).

### 3.1 Determinants of age‐inappropriate breastfeeding

After adjusting for other covariates, there was an association between age‐inappropriate breastfeeding and the age of the child. As seen in Table 2, children aged 0–5 months had a 70% greater odds of being age‐inappropriately breastfed, whereas children 6–17 months had approximately 50% lower odds of being age‐inappropriately breastfed compared with children aged 18–23 months. Children with siblings had approximately 30% lower odds of being age‐inappropriately breastfed compared with the first child.

**Table 2:** Determinants of age‐inappropriate breastfeeding in children aged 0–23 months (*N* = 11,687)

Factors	Univariable			Multivariable		
	Odds ratio	95% confidence interval	Wald test *P* value	Adjusted odds ratio	95% confidence interval	Wald test *P* value
Survey year			.30			.28
2012	Reference			Reference		
2017	0.95	[0.85, 1.05]		0.94	[0.84, 1.05]	
Child factors						
Gender			.05			
Male	Reference					
Female	0.91	[0.82, 1.00]				
Child's age			<.0001			<.0001
0–5 months	1.60	[1.39, 1.84]		1.70	[1.47, 1.97]	
6–8 months	0.47	[0.39, 0.56]		0.48	[0.40, 0.58]	
9–11 months	0.43	[0.36, 0.52]		0.43	[0.36, 0.51]	
12–17 months	0.49	[0.42, 0.57]		0.49	[0.42, 0.57]	
18–23 months	Reference			Reference		
Birth order			<.0001			
First child	Reference			Reference		<.0001
Second child	0.73	[0.64, 0.82]		0.69	[0.61, 0.78]	
3+	0.72	[0.64, 0.81]		0.70	[0.62, 0.80]	
Demographic factors						
Mother's age			.28			
15–19 years	1.14	[0.89, 1.45]				
20–24 years	1.16	[0.99, 1.35]				
25–29 years	1.09	[0.95, 1.24]				
30–34 years	Reference					
35–39 years	1.04	[0.88, 1.23]				
40+ years	0.91	[0.73, 1.14]				
Age difference between mother and father			.002			
Woman older than man	Reference					
0–4 years	0.92	[0.80, 1.05]				
5–7 years	0.83	[0.71, 0.96]				
>7 years	0.75	[0.64, 0.88]				
Father's educational attainment			<.0001			
Incomplete primary education/none	0.71	[0.59, 0.86]				
Complete primary or some secondary	0.68	[0.61, 0.75]				
Completed secondary or higher	Reference					
Father's occupation			.0001			
Agricultural	Reference					
Nonagricultural	1.22	[1.10, 1.35]				
Residence			<.0001			.008
Urban	Reference			Reference		
Rural	0.75	[0.68, 0.84]		0.85	[0.75, 0.96]	
Region			.05			<.0001
West	Reference			Reference		
East	1.10	[1.00, 1.20]		1.26	[1.14, 1.40]	
Household factors						
Wealth quintiles			<.0001			<.0001
Lowest	Reference			Reference		
Second	1.00	[0.85, 1.18]		1.05	[0.89, 1.24]	
Third	1.11	[0.96, 1.30]		1.20	[1.01, 1.43]	
Fourth	1.33	[1.14, 1.54]		1.34	[1.12, 1.61]	
Highest	1.61	[1.38, 1.89]		1.63	[1.33, 2.00]	
Number of means of transport owned			.03			
None	Reference					
One means	1.21	[1.05, 1.40]				
Two or more	1.21	[1.04, 1.41]				
Area of agricultural land			.05			
Did not own agricultural land	Reference					
<3 ha	0.92	[0.77, 1.10]				
3–9 ha	0.80	[0.68, 0.95]				
10+ ha	1.04	[0.92, 1.19]				
Number of children under five			.42			
<2	Reference					
Two or more	1.04	[0.94, 1.16]				
Health care factors						
Number of ANC visits	1.00	[0.98, 1.01]	.81			
Quality of ANC			.0012			<.0001
1–3 types of service	Reference			Reference		
4–6 types of service	0.87	[0.73, 1.05]		0.73	[0.60, 0.88]	
7–8 types of service	0.79	[0.65, 0.96]		0.63	[0.51, 0.77]	
ANC: had consultation and told about care for complication			.27			
Not had consultation nor told about care	Reference					
Had either one	1.12	[0.95, 1.32]				
Had both	1.14	[0.97, 1.35]				
Delivery assistant			.0009			
Untrained	Reference					
Trained	1.30	[1.11, 1.51]				
Women's empowerment factors^a^						
Mother's labour force participation			<.0001			
Low	Reference			Reference		<.0001
Medium	1.25	[1.10, 1.41]		1.12	[0.98, 1.28]	
High	1.69	[1.48, 1.92]		1.62	[1.41, 1.87]	
Mother's disagreement towards wife beating			.0002			
Low	Reference					
Medium	1.24	[1.10, 1.41]				
High	1.01	[0.89, 1.14]				
Mother's decision‐making power			.59			
Low	Reference					
Medium	0.95	[0.84, 1.07]				
High	1.01	[0.90, 1.14]				
Mother's knowledge level			.0002			
Low	Reference					
Medium	1.00	[0.89, 1.13]				
High	1.26	[1.11, 1.42]				

Abbreviation: ANC, antenatal care.

a Women's empowerment factors were calculated by combining the data of the two surveys and using principal component analysis of 17 indicators following a previous method (Sebayang et al., 2019). Six variables indicated labour force participation: work in the last 12 months, for whom the woman worked, women's occupation type, types of payment, worked all year, and earned more than the husband. Women's disagreement with justification for wife beating was assessed with five reasons: neglecting children, going out without husband's permission, arguing with husband, refusing sex, and burning food. Decision‐making power was derived from household decision makers for women's own health care, household purchases, visiting family, and husband's earnings. The women's knowledge component included formal educational level and access to media (newspaper, radio, and television).

Children living in rural areas had 15% lower odds of being age‐inappropriately breastfed compared with children living in urban areas. Children in the east of Indonesia had 26% higher odds of being age‐inappropriately breastfed compared with those in the west of Indonesia (Table 2). When we categorised Kalimantan in west Indonesia, the odds of age‐inappropriate breastfeeding for east Indonesia were 17% higher (odds ratio [OR] = 1.17, 95% CI [1.05, 1.30], *P* = .006) than that of west Indonesia. Children in Maluku had more than four times the odds of being age‐inappropriately breastfed compared with children in Yogyakarta (Figure S5).

There was an association between socio‐economic status and age‐inappropriate breastfeeding. Interestingly, compared with the lowest quintile, children from households in the higher quintiles, that is, the third, fourth, and highest quintiles had a 20%, 34%, and 63% greater odds of being age‐inappropriately breastfed, respectively. Children of mothers who received more types of services during ANC in her last pregnancy had lower odds of being age‐inappropriately breastfed.

In terms of women's empowerment, only mother's labour force participation was associated with age‐inappropriate breastfeeding. Children of mothers who had a high level of labour force participation had 62% higher odds of being age‐inappropriately breastfed (adjusted OR = 1.62, 95% CI [1.41, 1.87]; Table 2).

### 3.2 Determinants of dietary diversity

After controlling for child, demographic, and household factors, we found factors associated with an increased score of dietary diversity were the child's age, region, and wealth quintiles (Table 3). The older the child's age, the greater the dietary diversity score compared with children aged 6–8 months. Similarly, the higher the socio‐economic status of the household, the greater dietary diversity score of the child (Table 3). Children who lived in the east of Indonesia had a 0.22 lower dietary diversity score compared with children in the west of Indonesia (Table 3). When we included Kalimantan in west Indonesia, the dietary diversity score for east Indonesia was 0.29 lesser (95% CI [−0.38, 0.19], *P* < .0001) than that of west Indonesia. Children in West Papua had a dietary diversity score 1 point lower compared with those living in Yogyakarta (Figure S5).

**Table 3:** Determinants of dietary diversity in children aged 6–23 months (*N* = 8,878)

Factors	Univariable			Multivariable		
	Mean difference	95% confidence interval	Wald test *P* value	Adjusted mean difference	95% confidence interval	Wald test *P* value
Survey year			.88			.0001
2012	Reference			Reference		
2017	−0.01	[−0.11, 0.10]		−0.19	[−0.29, −0.10]	
Child factors						
Gender			.03			
Male	Reference					
Female	0.10	[0.01, 0.19]				
Child's age			<.0001			<.0001
6–8 months	Reference			Reference		
9–11 months	1.21	[1.05, 1.37]		1.21	[1.06, 1.37]	
12–17 months	1.66	[1.53, 1.80]		1.69	[1.56, 1.82]	
18–23 months	1.83	[1.69, 1.97]		1.83	[1.69, 1.97]	
Birth order			.004			
First child	Reference					
Second child	−0.05	[−0.17, 0.08]				
3+	−0.19	[0.31, −0.07]				
Demographic factors						
Mother's age			.01			
15–19 years	−0.44	[0.72, −0.16]				
20–24 years	−0.20	[0.35, −0.06]				
25–29 years	−0.14	[−0.27, −0.01]				
30–34 years	Reference					
35–39 years	−0.07	[−0.23, 0.08]				
40+ years	−0.18	[−0.37, 0.02]				
Age difference between mother and father			.04			
Woman older than man	Reference					
0–4 years	−0.02	[−0.16, 0.11]				
5–7 years	−0.03	[−0.18, 0.12]				
>7 years	−0.19	[−0.34, −0.03]				
Father's educational attainment			<.0001			
Incomplete primary education/none	−0.66	[−0.84, −0.48]				
Complete primary or some secondary	−0.40	[−0.51, −0.30]				
Completed secondary or higher	Reference					
Father's occupation			<.0001			
Agricultural	Reference					
Nonagricultural	0.36	[0.26, 0.46]				
Residence			<.0001			
Urban	Reference					
Rural	−0.43	[−0.53, −0.32]				
Region			<.0001			<.0001
West	Reference			Reference		
East	−0.42	[−0.51, −0.32]		−0.22	[−0.31, −0.13]	
Household factors						
Wealth quintiles			<.0001			<.0001
Lowest	Reference			Reference		
Second	0.49	[0.35, 0.64]		0.33	[0.19, 0.46]	
Third	0.66	[0.52, 0.81]		0.44	[0.29, 0.59]	
Fourth	0.83	[0.69, 0.97]		0.51	[0.36, 0.66]	
Highest	1.18	[1.04, 1.33]		0.73	[0.56, 0.90]	
Number of means of transport owned			<.0001			
None	Reference					
One means	0.49	[0.36, 0.62]				
Two or more	0.76	[0.61, 0.91]				
Area of agricultural land			<.0001			
Did not own agricultural land	Reference					
<3 ha	−0.20	[−0.38, −0.03]				
3–9 ha	−0.28	[−0.44, −0.11]				
10+ ha	−0.25	[−0.38, −0.13]				
Number of children under five			.01			
<2	Reference					
Two or more	−0.14	[−0.25, −0.04]				
Health care factors						
Number of ANC visits	0.09	[0.07, 0.10]	<.0001	0.03	[0.02, 0.04]	<.0001
Quality of ANC			<.0001			
1–3 types of service	Reference			Reference		<.0001
4–6 types of service	10.72	[0.55, 0.89]		0.24	[0.07, 0.41]	
7–8 types of service	1.07	[0.89, 1.24]		0.49	[0.30, 0.68]	
ANC: had consultation and told about care for complication			<.0001			
Not had consultation nor told about care	Reference			Reference		.008
Had either one	0.51	[0.35, 0.66]		0.09	[−0.06, 0.23]	
Had both	0.77	[0.61, 0.93]		0.22	[0.06, 0.37]	
Women's empowerment factors^a^						
Mother's labour force participation			<.0001			.0002
Low	Reference			Reference		
Medium	0.29	[0.18, 0.40]		0.08	[−0.03, 0.18]	
High	0.56	[0.44, 0.68]		0.24	[0.12, 0.36]	
Mother's disagreement towards wife beating			.39			
Low	Reference					
Medium	0.03	[−0.08, 0.15]				
High	0.08	[−0.03, 0.19]				
Mother's decision‐making power			.41			
Low	Reference					
Medium	−0.02	[−0.13, 0.10]				
High	0.06	[−0.06, 0.18]				
Mother's knowledge level			<.0001			.0002
Low	Reference			Reference		
Medium	0.25	[0.13, 0.37]		0.12	[0.01, 0.22]	
High	0.57	[0.44, 0.69]		0.26	[0.14, 0.38]	

Abbreviation: ANC, antenatal care.

a Women's empowerment factors were calculated by combining the data of the two surveys and using principal component analysis of 17 indicators following a previous method (Sebayang et al., 2019). Six variables indicated labour force participation: work in the last 12 months, for whom the woman worked, women's occupation type, types of payment, worked all year, and earned more than the husband. Women's disagreement with justification for wife beating was assessed with five reasons: neglecting children, going out without husband's permission, arguing with husband, refusing sex, and burning food. Decision‐making power was derived from household decision makers for women's own health care, household purchases, visiting family, and husband's earnings. The women's knowledge component included formal educational level and access to media (newspaper, radio, and television).

All health care factors were positively associated with the dietary diversity score. One ANC visit was associated with 0.03 more points of dietary diversity score, 4–6 types of ANC services were associated with 0.24 more points of dietary diversity score, and 7–8 types of ANC services were associated with 0.49 more points of dietary diversity score. Children of mothers who had both consultations during ANC and were told about care for complication had 0.22 more points of dietary diversity score compared with those who had neither one. Women's empowerment, as indicated by the mother's labour force participation and her knowledge level, were significant positive determinants of dietary diversity. Children of mothers with high labour force participation had 0.24 points higher dietary diversity score, whereas children of mothers with medium knowledge had 0.12 more points and those of mothers with high knowledge had 0.26 points higher dietary diversity score (Table 3).

### 3.3 Determinants of ASF

The child's age was a significant factor for adequate intake of ASF. The older the child, the greater the odds of consuming more types of ASF. Compared with children aged 6–8 months, children aged 9–11 months had more than three times greater odds of consuming 3+ types of ASF, children 12–17 months had nearly six times greater odds, and children 18–23 months had more than eight times greater odds of consuming 3+ types of ASF (Table 4).

**Table 4:** Determinants of consumption of 3+ types of animal source food in children aged 6–23 months (*N* = 8,878)

Factors	Univariable			Multivariable		
	Odds ratio	95% confidence interval	Wald test *P* value	Adjusted odds ratio	95% Confidence Interval	Wald test p‐value
Survey year			.08			.34
2012	Reference			Reference		
2017	1.11	[0.99, 1.25]		0.94	[0.82, 1.07]	
Child factors						
Gender			.11			
Male	Reference					
Female	1.10	[0.98, 1.23]				
Child's age			<.0001			<.0001
6–8 months	Reference			Reference		
9–11 months	3.46	[2.69, 4.45]		3.64	[2.81, 4.72]	
12–17 months	5.35	[4.26, 6.72]		5.87	[4.65, 7.42]	
18–23 months	7.34	[5.85, 9.20]		8.06	[6.39, 10.17]	
Birth order			.003			
First child	Reference					
Second child	0.98	[0.86, 1.13]				
3+	0.81	[0.71, 0.92]				
Demographic factors						
Mother's age			.002			
15–19 years	0.65	[0.47, 0.90]				
20–24 years	0.78	[0.66, 0.92]				
25–29 years	0.96	[0.82, 1.11]				
30–34 years	Reference					
35–39 years	0.94	[0.79, 1.12]				
40+ years	0.72	[0.57, 0.92]				
Age difference between mother and father			.002			
Women older than man	Reference					
0–4 years	0.97	[0.83, 1.14]				
5–7 years	0.87	[0.73, 1.04]				
>7 years	0.74	[0.62, 0.89]				
Father's educational attainment			<.0001			
Incomplete primary education/none	0.47	[0.38, 0.59]				
Complete primary or some secondary	0.62	[0.55, 0.70]				
Completed secondary or higher	Reference					
Father's occupation			<.0001			
Agricultural	Reference					
Nonagricultural	1.31	[1.16, 1.48]				
Residence			<.0001			
Urban	Reference					
Rural	0.63	[0.56, 0.71]				
Region			.0002			.53
West	Reference			Reference		
East	0.81	[0.73, 0.90]		0.96	[0.85, 1.09]	
Household factors						
Wealth quintiles			<.0001			<.0001
Lowest	Reference			Reference		
Second	1.52	[1.26, 1.84]		1.44	[1.16, 1.78]	
Third	1.89	[1.57, 2.27]		1.74	[1.41, 2.16]	
Fourth	2.40	[2.01, 2.87]		2.09	[1.68, 2.60]	
Highest	3.42	[2.84, 4.13]		2.79	[2.19, 3.56]	
Number of means of transport owned			<.0001			
None	Reference					
One means	1.56	[1.32, 1.85]				
Two or more	2.03	[1.70, 2.43]				
Area of agricultural land			<.0001			.003
Did not own agricultural land	Reference			Reference		
<3 ha	0.66	[0.53, 0.81]		0.72	[0.57, 0.91]	
3–9 ha	0.67	[0.55, 0.82]		0.85	[0.68, 1.06]	
10+ ha	0.90	[0.78, 1.03]		1.13	[0.97, 1.32]	
Number of children under five			.52			
<2	Reference					
Two or more	0.96	[0.86, 1.08]				
Health care factors						
Number of ANC visits	1.07	[1.05, 1.09]	<.0001			
Quality of ANC			<.0001			<.0001
1–3 types of service	Reference			Reference		
4–6 types of service	1.86	[1.50, 2.32]		1.41	[1.10, 1.79]	
7–8 types of service	2.45	[1.95, 3.07]		1.81	[1.40, 2.34]	
ANC: had consultation and told about care for complication			<.0001			
Not had consultation nor told about care	Reference					
Had either one	1.40	[1.16, 1.68]				
Had both	1.77	[1.48, 2.12]				
Women's empowerment factors^a^						
Mother's labour force participation			<.0001			.001
Low	Reference			Reference		
Medium	1.36	[1.18, 1.55]		1.08	[0.93, 1.26]	
High	1.77	[1.54, 2.03]		1.33	[1.13, 1.55]	
Mother's disagreement towards wife beating			.09			
Low	Reference					
Medium	1.12	[0.97, 1.30]				
High	1.16	[1.01, 1.34]				
Mother's decision‐making power			.96			
Low	Reference					
Medium	0.99	[0.86, 1.14]				
High	1.01	[0.88, 1.16]				
Mother's knowledge level			<.0001			.0001
Low	Reference			Reference		
Medium	1.27	[1.10, 1.46]		1.18	[1.01, 1.37]	
High	1.80	[1.56, 2.06]		1.43	[1.22, 1.69]	

Abbreviation: ANC, antenatal care.

a Women's empowerment factors were calculated by combining the data of the two surveys and using principal component analysis of 17 indicators following a previous method (Sebayang et al., 2019). Six variables indicated labour force participation: work in the last 12 months, for whom the woman worked, women's occupation type, types of payment, worked all year, and earned more than the husband. Women's disagreement with justification for wife beating was assessed with five reasons: neglecting children, going out without husband's permission, arguing with husband, refusing sex, and burning food. Decision‐making power was derived from household decision makers for women's own health care, household purchases, visiting family, and husband's earnings. The women's knowledge component included formal educational level and access to media (newspaper, radio, and television).

When we included Kalimantan in west Indonesia, the odds of children consuming 3+ types of ASF in east Indonesia were 21% (OR = 0.79, 95% CI [0.69, 0.90], *P* = .0004) lower compared with west Indonesia. Children in West Sulawesi had the lowest odds of consuming 3+ types of ASF with 70% lower odds compared with children in Central Kalimantan (Figure S5).

The significant household determinant was socio‐economic status. Children from the higher quintiles households had greater odds of consuming 3+ types of ASF. The highest odds were in children in the top wealth quintile, with nearly three times the odds of consuming 3+ types of ASF. Children of families owning <3 ha of agricultural land had 28% lower odds of consuming 3+ types of ASF. Quality of ANC was associated with ASF consumption with children of women who had 7–8 types of ANC services during her last pregnancy, having 81% greater odds of consuming 3+ types of ASF.

Children of women with high labour force participation had 33% higher odds of consuming 3+ types of ASF compared with children of women with low labour force participation, and children of women with a high knowledge level had 43% higher odds of consuming 3+ types of ASF compared with children of women with low knowledge level (Table 4).

## 4 DISCUSSION

### 4.1 General findings

In this study, age‐inappropriate breastfeeding, dietary diversity, and consumption of ASF were associated with the child's age, household socio‐economic status, quality of ANC, and labour force participation of mothers. Children aged 0–5 months and those whose mother was of higher socio‐economic status and higher labour force participation, had greater odds of age‐inappropriate breastfeeding. Higher birth order of the child and living in a rural area were associated with lower age‐inappropriate breastfeeding. We did not find any association between having means of transport as a proxy of access to markets with any of the outcomes. Land ownership as a proxy for food availability was only associated with lower ASF consumption. A larger number of ANC visits and whether women received both consultation and information about care for complications were associated with more dietary diversity, and better quality of ANC was associated with both more dietary diversity and variety of consumption of 3+ types of ASF in children. For women's empowerment, apart from women's labour force participation that was associated with all outcomes, high women's knowledge level was also associated with more dietary diversity and greater odds of consuming 3+ types of ASF among children.

### 4.2 Comparison with other studies

Similar to our findings for age‐inappropriate breastfeeding, a study using combined 2002/2003 and 2007 IDHS reported that being richer and having both parents working were associated with lower odds of exclusive breastfeeding (Titaley et al., 2014). In contrast, in Ethiopia, mother's education level and the number of ANC visits were determinants of exclusive breastfeeding (Tariku et al., 2017), although we did not find such an association for age‐inappropriate breastfeeding. In agreement with our results, a study in urban Indonesia found a positive association between minimum dietary diversity and socio‐economic status (Santika, Februhartanty, & Ariawan, 2016). A similar study using the IDHS 2007 data (Dominguez‐Salas, Cox, Prentice, Hennig, & Moore, 2012) found similar determinants for minimum dietary diversity, which included child age, household wealth, region, and women's educational indicators (mother's education and interaction with media), but urban/rural residence was not a factor for dietary diversity in our study. In India and East Africa, child's age, mother's education, household wealth, and the number of ANC visits were positively associated with dietary diversity (Gewa & Leslie, 2015; Patel et al., 2012). Similarly, a study in Ethiopia also found that the mother's education, occupation, and household income were positively associated with minimum dietary diversity (Solomon et al., 2017).

### 4.3 Possible mechanisms

The highest odds of age‐inappropriate breastfeeding were at 0–5 months, indicating that many children were not exclusively breastfed. Many mothers have stopped breastfeeding their babies at the age of 18–23 months. Several possible reasons for this included a perception of insufficient breast milk and influence from family members (Barati et al., 2018; Kavle, LaCroix, Dau, & Engmann, 2017). The lower odds in ASF consumption between ages 6–8 and 12–17 months indicate that many mothers may have waited until 18 months to introduce more types of ASF. The delay may be influenced by limited income and livestock for foods (Wong et al., 2018) that may result in the family's choice of prioritising priced foods only to productive adults but could also be influenced by the lack of caregiver's knowledge on the proper age to introduce more varieties of ASF other than milk to children. The pathway of association between socio‐economic status and our outcomes may be due to increased means to purchase baby formula in higher socio‐economic status families. Mothers in richer households, thus, weaned babies earlier and gave children more diversity of animal and plant source complementary food, whereas mothers in poorer households would delay feeding their babies more ASF, either because of costs or lack of knowledge. A study in urban Indonesia showed a similar trend in which children in lower socio‐economic households had higher odds of continued breastfeeding at 1 year and lower odds of early introduction to solid, semisolid, and soft foods at age 6 months (Santika et al., 2016). Similarly, mothers with high labour force participation may have greater means to purchase baby formula and less time to breastfeed or express their milk for later use, hence, wean their babies earlier (Barati et al., 2018). Mothers seemed to be better at age‐appropriate breastfeeding when babies were the second or later child, possibly due to limited funds to purchase formula for the last child as the total cost to raise children increases with each additional child. Agricultural families who only have a small size of land may not have enough animal products for consumption and thus prioritise selling the ASF rather than their family consuming it (Wong et al., 2018).

Quality of ANC was a proxy for access to health information that may improve the likelihood that mothers or caregivers receive information about proper child feeding. Having consultation during ANC, however, was only associated with improved dietary diversity but not with other outcomes, indicating that other factors, such as economy, were associated more with decisions about prolonging breastfeeding and feeding children more types of ASF. Consultation during ANC alone could not have addressed these issues; thus, health care providers should continue to give support for appropriate feeding practice to women, either through consultation or counselling until the child reaches 2 years of age. An Indonesian study found that although the majority of midwives (96%) reported discussing with pregnant women and caregivers about breastfeeding, only 70% discussed about exclusive breastfeeding until 6 months and 25% discussed prolonging breastfeeding until 2 years old (Beatty, Ingwersen, & Null, 2017). This finding indicates the need to revamp the child feeding consultation programme in Indonesia. Also, a study in Indonesia has reported that mothers who received free samples of infant formula in their delivery clinics mentioned that they continued giving formula to their children afterwards (Barati et al., 2018). This observation raises an important issue of the need to include marketing ethics in the measurement of health care quality.

### 4.4 Strengths and limitations

In this study, we combined two nationally representative data sets and, thus, had a large sample size to measure the determinants of child feeding in Indonesia. We used a stricter significance level to determine an association in order to reduce the possibility of associations by chance due to the large sample size. However, this study was cross‐sectional in design, and thus, we cannot infer causal relationships. The data sets did not include questions about food security and beliefs around child feeding, and thus, we could not assess the possibility that these factors predict child feeding practices. We also did not have a better measure of the mother's participation at postnatal care, which would have been a better proxy for her contact with health service.

### 4.5 Policy implications and future research

Our results show that the Indonesian government needs to focus on programmes to improve child feeding in all eastern parts of Indonesia as well as in several provinces in western Indonesia that were among in the worst half of the provinces. The provinces include Aceh, North Sumatera, and Riau for dietary diversity; Riau Islands, Bangka Belitung, Jakarta, Riau, Banten, and East Java for age‐inappropriate breastfeeding; and Bengkulu, North Sumatera, Central Java, West Java, and Aceh for the consumption of ASFs. Additionally, programmes need to focus on improving dietary diversity and consumption of ASFs for poorer households and on prolonging breastfeeding for richer households. Mother's participation in the labour force may improve dietary diversity and consumption of ASFs, and a previous study also shows that it improves ANC visits (Sebayang et al., 2019), which may eventually increase women's access to sound child feeding information and support. Women's labour force participation thus needs to be encouraged, but better national programmes are needed to support prolonged breastfeeding and to provide facilities for expressing breast milk for working mothers. Some studies have shown an impact of maternity leave and workplace support programmes on improving exclusive breastfeeding, but studies on the impact on continued breastfeeding are lacking (Rollins et al., 2016). In Indonesia, the quality of consultation in ANC and postnatal care programmes needs improvement and should cover both breastfeeding and complementary feeding practices, food diversity, and the economic benefits of appropriate child feeding. Antenatal counselling programmes are reported to increase exclusive breastfeeding in children under 6 months by 66% and continued breastfeeding in children aged 12–23 months by 15% (Rollins et al., 2016). A recent review of seven studies in south Asia showed that information, education, and counselling interventions improved dietary diversity (Aguayo, 2017). In Indonesia, providing community activation through women's groups, in addition to general nutrition information, improved exclusive breastfeeding in the first 3 months of life by 16% (S. White et al., 2016). Programmes to enhance the delivery of nutrition information through women's groups need promoting in Indonesia.

## CONFLICTS OF INTEREST

The authors declare that they have no conflicts of interest.

## CONTRIBUTIONS

SKS conceptualised the study, conducted the data analysis, and drafted and revised the manuscript. MJD conceptualised the study, contributed to the data analysis, and revised the manuscript. E A contributed to the data analysis and revised the manuscript. FE contributed to conceptualising the study and reviewed the manuscript. PJK contributed to the data analysis and reviewed the manuscript. ML contributed to conceptualising the study and reviewed the manuscript.

## Supporting information

Figure S1. Analytical Framework of Determinants of Age‐inappropriate breastfeeding, Dietary Diversity Score and Consumption of 3+ Types of Animal Source Foods

Figure S2. Percentage of age‐inappropriate breastfeeding in children age 0 – 23 months by province in a) 2012 and b) 2017

Figure S3. Mean dietary diversity in children age 6 – 23 months in a) 2012 and b) 2017

Figure S4. Percentage of 3+ types of animal source food consumption in children age 6 – 23 months by province in a) 2012 and b) 2017

Figure S5. Adjusted Odds Ratio of age‐inappropriate breastfeeding in children 0‐23 months (N=11687), adjusted means of dietary diversity in children 6‐23 months (N=8878) and adjusted Odds Ratio of 3+ types of animal source food consumption in children 6‐23 months (N=8878) by province
