# Supplementary material for: Determinants of age‐appropriate breastfeeding, dietary diversity, and consumption of animal source foods among Indonesian children
Source: Matern Child Nutr. 2019 Oct 2;16(1):e12889. doi: 10.1111/mcn.12889 (PMC7038882; doi:10.1111/mcn.12889)
Supplement: Supplementary file 1 — Figure S1. Analytical Framework of Determinants of Age‐inappropriate breastfeeding, Dietary Diversity Score and Consumption of 3+ Types of Animal Source Foods [file MCN-16-e12889-s001.docx]

Supplementary File 1. Analytical Framework of Determinants of Age-inappropriate breastfeeding, Dietary Diversity Score and Consumption of 3+ Types of Animal Source Foods

**Outcome: Child Feeding**

Age-inappropriate Breastfeeding

Dietary Diversity Score

Consumption of 3+ Types of Animal Source Foods

**Child Factors:**

Gender

Child’s age

Birth order

**Demographic Factors:**

Mother’s age

Age difference between mother & father

Father’s education

Father’s occupation

Residence

Region

**Household Factors:**

Wealth quintiles

Number of means of transport owned

Size of agricultural land owned

Number of children under 5

**Health Care Factors:**

Number of antenatal care (ANC) attended

Quality of ANC

ANC: had consultation and told about care for pregnancy complication

Delivery assisted by trained professional (for age-inappropriate breastfeeding only)

**Women’s Empowerment Factors:**

Labour force participation

Disagreement to justification towards wife beating

Decision-making power

Women’s knowledge level
