# Supplementary material for: Determinants of age‐appropriate breastfeeding, dietary diversity, and consumption of animal source foods among Indonesian children
Source: Matern Child Nutr. 2019 Oct 2;16(1):e12889. doi: 10.1111/mcn.12889 (PMC7038882; doi:10.1111/mcn.12889)

Supplementary File 2. Percentage of age-inappropriate breastfeeding in children age 0 – 23 months by province in a) 2012 and b) 2017


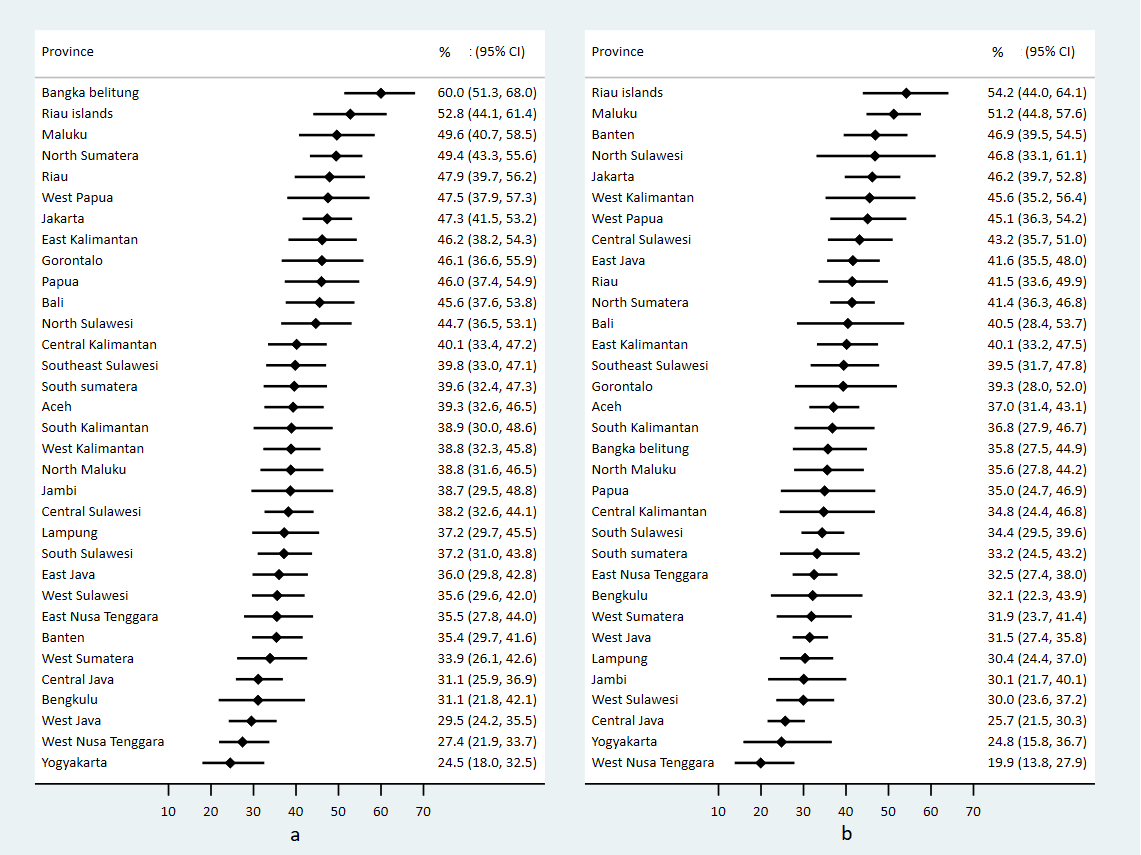

Supplement: Supplementary file 2 — Figure S2. Percentage of age‐inappropriate breastfeeding in children age 0 – 23 months by province in a) 2012 and b) 2017 [file MCN-16-e12889-s002.docx]
