# Supplementary figures and images for: Determinants of age‐appropriate breastfeeding, dietary diversity, and consumption of animal source foods among Indonesian children
Source: Matern Child Nutr. 2019 Oct 2;16(1):e12889. doi: 10.1111/mcn.12889 (PMC7038882; doi:10.1111/mcn.12889)

Supplementary File 3. Mean dietary diversity in children age 6 – 23 months in a) 2012 and b) 2017


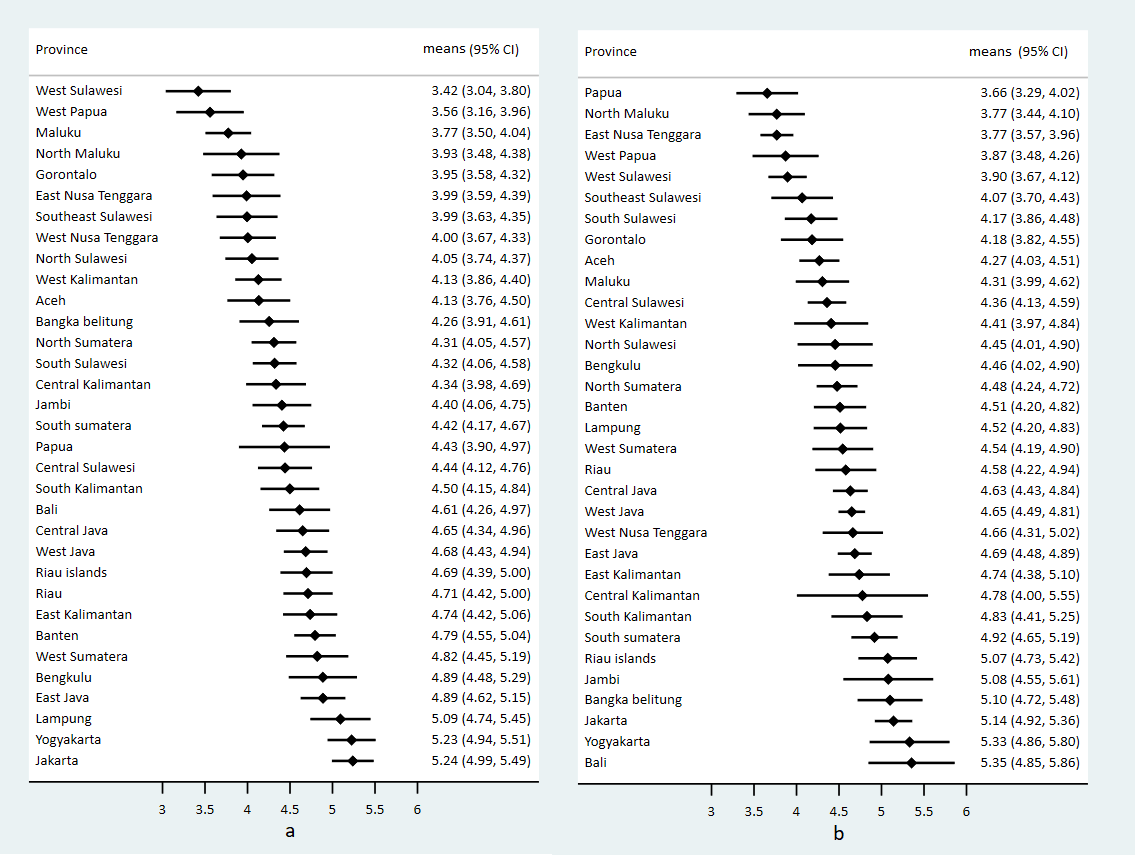

Supplement: Supplementary file 3 — Figure S3. Mean dietary diversity in children age 6 – 23 months in a) 2012 and b) 2017 [file MCN-16-e12889-s003.docx]
