# Supplementary material for: Determinants of age‐appropriate breastfeeding, dietary diversity, and consumption of animal source foods among Indonesian children
Source: Matern Child Nutr. 2019 Oct 2;16(1):e12889. doi: 10.1111/mcn.12889 (PMC7038882; doi:10.1111/mcn.12889)
Supplement: Supplementary file 5 — Figure S5. Adjusted Odds Ratio of age‐inappropriate breastfeeding in children 0‐23 months (N=11687), adjusted means of dietary diversity in children 6‐23 months (N=8878) and adjusted Odds Ratio of 3+ types of animal source food consumption in children 6‐23 months (N=8878) by province [file MCN-16-e12889-s005.docx]

Supplementary File 5. Adjusted Odds Ratio of age-inappropriate breastfeeding in children 0-23 months (N=11687), adjusted means of dietary diversity in children 6-23 months (N=8878) and adjusted Odds Ratio of 3+ types of animal source food consumption in children 6-23 months (N=8878) by province

|  | Age-inappropriate breastfeeding | | Dietary Diversity | | 3+ types of Animal Source Food | |
| --- | --- | --- | --- | --- | --- | --- |
| Province | Adj Or^a^ | 95% Confidence Interval | Adj Means^b^ | 95% Confidence Interval | Adj OR^c^ | 95% Confidence Interval |
| Aceh | 2.09 | 1.37 - 3.19 | -0.70 | -0.98 - -0.42 | 0.52 | 0.32 - 0.86 |
| North Sumatera | 2.82 | 1.86 - 4.26 | -0.37 | -0.64 - -0.09 | 0.56 | 0.34 - 0.90 |
| West Sumatera | 1.70 | 1.06 - 2.72 | -0.27 | -0.58 - 0.04 | 0.60 | 0.34 - 1.03 |
| Riau | 2.58 | 1.63 - 4.08 | -0.26 | -0.57 - 0.05 | 0.60 | 0.37 – 1.00 |
| Jambi | 1.82 | 1.12 - 2.98 | -0.15 | -0.50 - 0.19 | 0.64 | 0.38 - 1.09 |
| South sumatera | 1.75 | 1.10 - 2.78 | -0.30 | -0.60 – 0.00 | 0.69 | 0.41 - 1.16 |
| Bengkulu | 1.60 | 0.97 - 2.65 | -0.24 | -0.58 - 0.11 | 0.49 | 0.29 - 0.84 |
| Lampung | 1.90 | 1.21 - 2.98 | -0.12 | -0.44 - 0.20 | 0.72 | 0.43 - 1.21 |
| Bangka belitung | 2.74 | 1.68 - 4.47 | -0.43 | -0.76 - -0.11 | 0.82 | 0.46 - 1.45 |
| Riau islands | 3.49 | 2.17 - 5.63 | -0.30 | -0.62 - 0.02 | 0.75 | 0.43 - 1.29 |
| Jakarta | 2.50 | 1.65 - 3.78 | -0.20 | -0.47 - 0.06 | 0.86 | 0.52 - 1.42 |
| West Java | 1.40 | 0.93 - 2.10 | -0.48 | -0.73 - -0.22 | 0.42 | 0.26 - 0.67 |
| Central Java | 1.24 | 0.82 - 1.87 | -0.51 | -0.78 - -0.24 | 0.42 | 0.26 - 0.68 |
| Yogyakarta | Reference |  | Reference |  | 0.65 | 0.37 - 1.15 |
| East Java | 1.95 | 1.29 - 2.96 | -0.27 | -0.53 – 0.00 | 0.52 | 0.32 - 0.86 |
| Banten | 2.11 | 1.37 - 3.23 | -0.41 | -0.70 - -0.12 | 0.51 | 0.31 - 0.86 |
| Bali | 1.93 | 1.21 - 3.08 | -0.24 | -0.60 - 0.13 | 0.73 | 0.40 - 1.34 |
| West Nusa Tenggara | 1.12 | 0.71 - 1.78 | -0.70 | -1.01 - -0.38 | 0.37 | 0.22 - 0.62 |
| East Nusa Tenggara | 2.28 | 1.45 - 3.58 | -0.77 | -1.06 - -0.48 | 0.42 | 0.25 - 0.70 |
| West Kalimantan | 2.73 | 1.74 - 4.30 | -0.54 | -0.86 - -0.22 | 0.87 | 0.52 - 1.45 |
| Central Kalimantan | 2.41 | 1.49 - 3.90 | -0.28 | -0.70 - 0.13 | Reference |  |
| South Kalimantan | 2.17 | 1.35 - 3.49 | -0.43 | -0.75 - -0.10 | 0.59 | 0.34 - 1.02 |
| East Kalimantan | 2.56 | 1.65 - 3.98 | -0.35 | -0.65 - -0.06 | 0.60 | 0.35 - 1.03 |
| North Sulawesi | 3.60 | 2.20 - 5.90 | -0.75 | -1.04 - -0.45 | 0.38 | 0.22 - 0.66 |
| Central Sulawesi | 2.57 | 1.67 - 3.96 | -0.44 | -0.73 - -0.16 | 0.60 | 0.35 - 1.00 |
| South Sulawesi | 1.98 | 1.30 - 3.02 | -0.71 | -1.00 - -0.43 | 0.40 | 0.24 - 0.65 |
| Southeast Sulawesi | 2.35 | 1.50 - 3.68 | -0.83 | -1.17 - -0.50 | 0.43 | 0.26 - 0.73 |
| Gorontalo | 3.03 | 1.84 - 4.99 | -0.78 | -1.11 - -0.45 | 0.46 | 0.26 - 0.80 |
| West Sulawesi | 1.91 | 1.24 - 2.96 | -0.99 | -1.27 - -0.71 | 0.30 | 0.17 - 0.50 |
| Maluku | 4.27 | 2.73 - 6.68 | -0.61 | -0.92 - -0.30 | 0.43 | 0.26 - 0.70 |
| North Maluku | 2.36 | 1.50 - 3.72 | -0.81 | -1.16 - -0.45 | 0.33 | 0.19 - 0.57 |
| West Papua | 3.29 | 2.09 - 5.18 | -1.01 | -1.34 - -0.68 | 0.33 | 0.19 - 0.60 |
| Papua | 2.64 | 1.58 - 4.40 | -0.56 | -0.93 - -0.20 | 0.35 | 0.17 - 0.70 |
| a Adjusted for survey year, child's age, birth order, residence, household wealth, ANC quality, and women's labour force participation | | | | | | |
| b Adjusted for survey year, child's age, household wealth, number of ANC visits, ANC quality, ANC consultation, women's labour force participation, and women's education and knowledge level | | | | | | |
| c Adjusted for survey year, child's age, household wealth, agricultural land ownership, ANC quality, women's labour force participation, and women's education and knowledge level | | | | | | |
